# Health financing reforms in the quest for universal health coverage: Challenges and opportunities in the context of Nepal

**DOI:** 10.7189/jogh.13.03021

**Published:** 2023-04-28

**Authors:** Achyut R Pandey

**Affiliations:** HERD International, Kathmandu, Nepal

Universal health coverage (UHC) has been a key topic for the global agenda for some time now, postulating that everyone should have access to essential medical care, regardless of their socioeconomic situation and without being exposed to extreme health expenditures [1]. UHC encompasses three dimensions – population coverage, service coverage, and cost coverage.

Nepal has been striving to bring UHC to its citizens, but is finding that it comes with a great cost; as related health care services frequently require higher resources, some other sectors may be given lower priority due to resource limitations. Here we examine the opportunities and challenges in health financing reforms in the context of Nepal’s pursuit of UHC.

## UHC IN NEPAL: WHERE DO WE STAND?

The World Health Organization estimated Nepal had a UHC service coverage index of 53.0% in 2019. Similarly, the UHC service capacity sub-index was estimated at 30.0%, while the sub-indices for non-communicable disease (NCD), infectious disease, and reproductive, maternal, newborn, and child health service coverages were 58.0%, 60.0%, and 77.0%, respectively [2]. According to the Institute for Health Metrics and Evaluation, Nepal’s UHC service coverage index (converted on a scale from 0 to 100) could reach 57.3% by 2030 [3]. The data indicate the need for rapid improvement in service capacity and NCDs service coverage sub-index.

## KEY CHALLENGES

### Changing pattern of diseases

**Figure 1:**
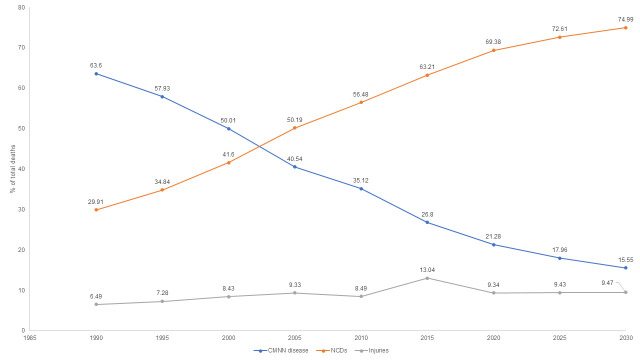
Changing pattern of disease mortality from 1990 to 2030.

As shown in **Figure 1**, the pattern of diseases has changed substantially over the past decades, with NCDs emerging as the primary burden. NCDs claimed a third of total deaths (29.91%) in 1990, which increased to 69.4% in 2020, and is anticipated to increase to 74.9% in 2030 [3]. As NCDs contribute majorly to out-of-pocket (OOP) expenditure [4], reducing such expenses requires increased investment in NCD care. According to a study done in Nepal, the adoption of several cost-effective strategies for NCDs would require an expense of US$8.76 per person. These initiatives, which would represent 22% of the current total health care spending, might be vital in attaining UHC by 2030 [5]. Moreover, NCDs require chronic care, demanding significant reorientation in the existing health system. One of the challenges for Nepal’s path to UHC will be to increase the budget on NCDs without deprioritizing communicable, maternal, neonatal, and nutritional (CMNN) diseases, which consume most of the current budget.

### Demand generation and population coverage

There could be additional 113 559 000 outpatient visits and 2 207 000 inpatient admission in Nepal if service utilization is increased to a level that meets the UHC target [6]. In Nepal, people typically wait to seek medical attention until their condition has considerably worsened. This could be due to increased adaptation to living with a disease or its symptoms, distrust of health facilities, a preference for traditional healing methods, reliance on home remedies, and perception that modern health care is costly [7]. Although substantial progress has been made in service expansion, generating demand in the community and increasing service utilization may continue to be a challenge in the upcoming days. Apart from infrastructure, ensuring an adequate number of skilled health workers to address additional health facility visits could be a daunting task for Nepal.

### Managing additional resource need

To achieve the UHC service utilization standard in 2016, an additional amount of 2.636 billion international dollars, accounting for approximately 3.50% of Nepal’s gross domestic product (GDP), is required [6], which could be challenging considering the size of the Nepalese economy. The situation could be further complicated by the decline in economic growth due to the COVID-19 pandemic. Taxation could be an option for generating additional revenue for the health system. However, increasing consumption taxes by a notable proportion could reduce the ability of the destitute population to aﬀord essential goods which could further impact their health status. For example, one study suggests that a US$100 increase in consumption taxes per person reduces the ability of the destitute to aﬀord necessities, and potentially increases the post-neonatal mortality rate by 0.17 points and under-five mortality rate by 0.43 points [8]. Nepal needs to strategically balance between the need to increase investment in health and additional revenue generation through taxation.

### Limited financial protection

OOP stands at 57.4% of the current health expenditure in Nepal [4], which needs to be reduced to around 15.0%-20.0% to maximally limit financial catastrophe and medical impoverishment [9]. In Nepal, 3.3% of the population spends more than 25% of its income on health, while 27.4% spends more than 10% of income on health [1]. Despite multiple policy interventions like the social security fund, free basic health care services, chronic disease support programme, safe motherhood incentives, free newborn care package, impoverished citizens’ programme, and national health insurance scheme, Nepal has not been able to reduce the proportion of OOP, and it remains a significant obstacle on the way to UHC.

**Figure:**
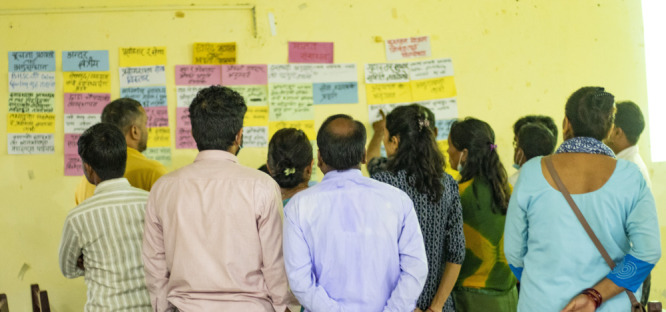
Photo: A group of health workers and researchers reviewing the list of interventions and deciding priorities for action in the Kapilvastu municipality of the Lumbini Province, Nepal. Source: HERD International, used with permission.

### The gap in quality of service

Kruk et al. estimated that 27 541 deaths could have been prevented in Nepal by population-level interventions, while another 46 400 deaths could have been averted through better health care services in 2019. Around 57.0% of all the deaths preventable through health care could be averted by improving the quality of service and the remaining 43.0% by increasing service utilization [10]. In 2019, approximately 1 114 000 years of life were lost due to poor quality of service in Nepal [10]. Together with the expansion of service, these findings point out the need of improving the quality of service.

### Persisting inequalities

There are persistent inequalities in service utilization and financial risk protection based on various parameters like geography, economic status, education, and ethnicity [11,12]. A segment of the population may be facing marginalization which has not been sufficiently addressed by current health policies. A national health insurance scheme can be an option to reduce OOP and prevent catastrophic health expenditure and medical impoverishment, particularly among the destitute and underprivileged population. Although Nepal has a provision that waives the premium for enrolment in the national health insurance scheme for poor citizens, identifying the impoverished has been a difficult task. Furthermore, as people may enter or exit poverty based on their income, regular reassessment of household poverty levels will continue to be a challenge.

## OPPORTUNITIES

### Expanding coverage of national health insurance scheme

Extending coverage of national health insurance schemes to include more of the country's underprivileged and poor population might lower the financial burden and medical impoverishment in Nepal. With a higher coverage level, the country can also include more services under the benefit package thereby reducing the OOP. Evidence suggests that, if pooled financial resources for health per person are increased by 10%, the UHC index value may improve by 1.4% [13].

A sizable number of dropouts in Nepal is endangering the long-term viability of its health insurance scheme. Potential remedies for this problem include digitizing the claims and reimbursement process, increasing the benefits package, implementing cost-sharing or co-payment models, providing top-ups in the benefits package, and providing subsidies during renewal if the insured person did not utilize health services in the previous year [14]. These steps might increase confidence and broaden health insurance coverage in Nepal. Apart from health insurance, Nepal has different social security schemes such as social security fund, chronic disease support programme, safe motherhood incentives, free newborn care, and others, which may have to be integrated and harmonized with national health insurance schemes.

### Improving efficiency within the health system

A previous study estimated that between 2015 and 2030, an increase in pooled resources could contribute to 58.1% of the improvement in the UHC index in low- and middle-income countries (LMICs). Additionally, 41.9% of the gains in the UHC index could result from enhancing efficiency within the health system and other contextual factors [13]. Since there has been slower growth in pooled resources in low-income countries, including Nepal, a larger fraction of UHC gains should be ensured through improvement in efficiency within the health system, which can bring universal health care closer, even if resource expansion is slow. The federal structure of Nepal, with seven provincial and 753 local governments, provides an opportunity to closely monitor the health system performance and seek cost-effective resource allocation based on locally available evidence. Nepal could consider shifting from a blanket approach of providing financial incentives to all, as in the Safe Motherhood Programme, to a more targeted approach that focuses on providing incentives specifically to the destitute and underprivileged population. This would allow for the more effective use of limited resources, ensuring that those who are most in need of assistance receive it, while minimizing the risk of resources being misused or wasted. Onboarding private hospitals in the delivery of service under a free newborn care package could help expand the service across the country.

### Contextualized intervention in a federal setting

Currently, the authority for delivering basic health care services has been devolved to local governments. This devolution could be an opportunity for contextualized planning for disease prevention, overcoming barriers to service utilization and reducing financial burden. With the present federal system of Nepal, areas such as agriculture, water supply, education, and infrastructure development fall under municipal offices. This may present an opportunity to establish preventative measures that are more likely to work in concert. For instance, extending the road network to improve access to medical facilities might aid in extending the service area. Waterborne infections may be less common if access to clean drinking water and sanitary facilities is improved. Actions to boost food production might help reduce the risk of malnutrition and promote food security.

### Research guided priority areas and promotion of frugal innovations

To reduce the financial burden on citizens, Nepal needs to regularly re-evaluate the burden of diseases, assess the cost-effectiveness of interventions and allocate more resources to low-cost high-impact interventions. This requires a substantial level of data on disease patterns as well as costs. The current federal structure in Nepal with 753 local governments, can be an opportunity to generate a pool of local and context-specific data to facilitate evidence-based decision-making. Embedding research within decision-making structures of the government and non-governmental sector could facilitate knowledge co-production and capacity transfer, ultimately bringing together the two worlds of research and policymaking together. Similarly, the adoption of technologies like telemedicine could help expand the quality of service to geographically remote areas.

## CONCLUSIONS

Considering the level of progress made in the past decades, Nepal’s path to UHC seems neither easy nor close. Further investments needed to achieve UHC could largely exceed the existing investment capacity of the country. Additional resource generation through expansion of national health insurance, improving efficiency within the health system, developing locally tailored intervention, evidence-based decision making, and promotion of frugal innovation could be strategies Nepal needs to harness to reach UHC by 2030.
